# Recurring Grippe

**Published:** 1894-01

**Authors:** I. N. Love

**Affiliations:** Vice-President American Medical Association


### RECURRING GRIPPE.

I. N. LOVE, M. D., VICE-PRESIDENT AMERICAN MEDICAL ASSOCIATION.

The history of epidemics is almost uniform in the direction of their extending over several years. Frequently the disease is endemic, becoming a definite part of every day life, as witness diphtheria in many sections of the country. LaGrippe is no exception. Appearing among us as it did several years ago, it returned the second year in a form more virulent than the first, producing effects far-reaching and uniformly demoralizing. Observing practitioners cannot have failed to notice that during this summer and fall many cases could be explained by no other hypothesis than that they were effected either directly or remotely to the grippe. The possibilities are that the coming winter and spring will develop enormous numbers of these cases; cases effectəd de novo by the germ—if there be one—and cases that have never yet recovered from previous attacks with re-aroused disturbances due to the sudden and frequent changes of the weather.

Feeling the importance of keeping open the excretory system of glands and at the same time considering thooughtfully the rheumatic feature that accompanies these cases, no remedy would more promptly suggest itself to our mind that of Tongaline. Knowing as we do definitely the component parts, the combination naturally suggests antagonism to a lock-up condition of the glands, opposition to neuralgia, rheumatism, nervous headache and gout.

We commend it earnestly and emphatically to the practitioners of the country at large to meet the conditions to which we have referred.

